# Thermal tolerance and acclimation limits in bull trout (*Salvelinus confluentus*) alevin

**DOI:** 10.1111/jfb.70580

**Published:** 2026-07-15

**Authors:** Brendan Maldonado, Travis Durhack, Andrew Chapelsky, Sarah E. Glowa, Analisa Lazaro‐Côté, Raven M. Bennett, Timothy Bartley, Neil J. Mochnacz

**Affiliations:** ^1^ Fisheries and Oceans Canada Winnipeg Manitoba Canada; ^2^ Department of Integrative Biology University of Guelph Guelph Ontario Canada; ^3^ Department of Biological Sciences University of Manitoba Winnipeg Manitoba Canada

**Keywords:** alevin, bull trout, climate warming, cold‐water habitat, critical thermal maximum, thermal tolerance

## Abstract

The critical thermal maximum (*CT*_max_) of bull trout (*Salvelinus confluentus*) alevin was measured after 3 weeks of acclimation to 4–21°C. *CT*_*max*_ increased from 24.3 at 4°C to 26.7 at 9°C, plateaued between 9 and 12°C (26.9°C), and declined to 25.9°C at 15°C. Acclimation mortality increased above 12°C, reaching 53.7% at 15°C, and no fish survived at 18–21°C. Condition factor declined at ≥12°C. Results indicate bull trout alevin have limited acclimation capacity beyond 12°C and suggest high vulnerability to lethal stress in streams where temperatures exceed this threshold.

Climate warming is a globally pervasive stressor that threatens the survival of many freshwater organisms (Barbarossa et al., 2021; Woodward et al., 2010). Ectotherms, such as freshwater fishes, are especially vulnerable because their body temperature is governed by the environment in which they reside (McDonnell & Chapman, 2015). Rising water temperatures may exceed their thermal limits, causing range shifts, physiological decline, or mortality when cooler refugia are not accessible (Roberts et al., 2013; Thurman et al., 2020). Predicting species' persistence under warming requires understanding thermal tolerance and acclimation capacity (i.e., acclimation potential; Sunday et al., 2010) across life stages. Therefore, evaluating a species' thermal tolerance and acclimation limits is critical for predicting how freshwater biodiversity will respond to climate warming.

Physiological performance across ecologically relevant temperatures can reveal thermal tolerance and acclimation potential in freshwater fishes (Comte & Olden, 2017; Li et al., 2021). Fishes vary in their ability to tolerate thermal stress, with some demonstrating greater phenotypic plasticity than others (Molina et al., 2024) and intraspecific variation occurring across populations (McKenzie et al., 2021). Thermal tolerance and acclimation capacity also differ across life stages, with early developmental stages, such as alevin, exhibiting narrower tolerances than older life stages (Dahlke et al., 2020), though there is active debate on this topic (See Dahlke et al., 2022; Pottier et al., 2022). Not only are early life stages generally understood to be more sensitive to temperature and other environmental fluctuations than older life stages, they also lack the mobility to avoid especially warm or hypoxic habitats (Del Rio et al., 2019). The alevin stage is an early post‐hatch developmental phase in fish during which the yolk sac remains attached, providing the primary source of nutrition until the onset of active feeding. Despite this sensitivity, few studies have examined thermal limits in salmonid alevin (Del Rio et al., 2019), yet understanding these limits is essential for predicting population responses to climate warming.

Critical thermal maximum (*CT*_max_) is a metric used to inform the upper thermal tolerance and acclimation capacity of fishes (Desforges et al., 2023). *CT*_max_ is quantified by gradually increasing water temperature until an organism loses equilibrium and essential functions, such as feeding and movement (Bates & Morley, 2020). Comparing *CT*_max_ across ecologically relevant acclimation temperatures reveals where thermal tolerance plateaus and declines. A plateau indicates physiological capacity limitations for further acclimation, while a decline marks the onset of physiological failure and potential mortality (Mayer et al., 2024).

Bull trout *Salvelinus confluentus* (Suckley 1859) inhabit cold‐water ecosystems in western North America and are of conservation concern due to their habitat specialization, late maturity, and infrequent spawning (Al‐Chokhachy et al., 2010). Consequently, populations across most of the Canadian distribution are listed as Threatened or Special Concern under the *Species at Risk Act* (COSEWIC, 2012). Bull trout from emergence to 1 year exhibit low thermal tolerance and reduced physiological function at temperatures between 12 and 18°C, with feed consumption and feed efficiency decreasing at temperatures above 14–16°C (McMahon et al., 2007; Selong et al., 2001). In the wild, populations rarely occur where summer stream temperatures exceed 11°C (Isaak et al., 2015; Isaak et al., 2017; Mochnacz et al., 2023). While juvenile and young‐of‐the‐year (YOY) thermal limits have been studied (Best et al., 2025; Selong et al., 2001), little is known about the upper thermal limits of the alevin stage. Previous research shows that *CT*_max_ increases with acclimation temperature until plateauing near 18°C in juvenile bull trout (Best et al., 2025) and 20°C in juvenile brook trout, a closely related species (Morrison et al., 2020). We aimed to estimate bull trout alevin *CT*_max_ across acclimation temperatures to assess upper sublethal tolerance and acclimation capacity. We predicted bull trout would exhibit a lower and narrower *CT*_max_ plateau than brook trout because they occupy a colder thermal niche (Isaak et al., 2017; Mochnacz et al., 2023). These findings will help clarify early‐life thermal sensitivity and inform conservation strategies for this threatened species.

On September 15, 2023, eggs and milt were collected from a wild bull trout population in Smith‐Dorrien Creek, Alberta, Canada, and transported by vehicle (15 h), in a temperature‐controlled environment with supplemental oxygen, to the Freshwater Institute Animal Holding Facility in Winnipeg, Manitoba, Canada. Fertilization occurred on September 16, 2023 by crossing 1 female with 1 male resulting in eight families. Fertilized eggs were separated by family into screened containers, placed in a Heath tray within a vertical‐flow stack incubator (Legend Brands Inc., Burlington, WA, USA), and incubated at 4°C with a recirculated water system. Embryos hatched after 86 days on December 11, 2023. Egg and alevin mortalities were periodically removed from each container using a pipette. Cyclic overhead lighting was adjusted monthly to simulate natural light conditions and the colour/intensity of sunrise/sunset for Smith‐Dorrien Creek.

*CT*_max_ trials were carried out from March 15, 2024 to April 25, 2024. Trials were conducted at 4°C (136 days post‐hatch; April 25, 2024), 6°C (121 days post‐hatch; April 10, 2024), 9°C (124 days post‐hatch; April 13, 2024), 12°C (127 days post‐hatch; April 16, 2024), 15°C (130 days post‐hatch; April 19, 2024) 18°C (130 days post‐hatch; April 19, 2024), and 21°C (130 days post‐hatch; April 19, 2024). To reach temperatures >6°C, bull trout were housed in incubator trays, which were warmed at a rate of 1°C/day from 4 to 8°C, then moved to treatment tanks to continue heating at the same rate until the treatment temperatures were reached. Fish were acclimated to their respective treatment temperatures for 3 weeks prior to *CT*_max_ trials. Acclimation for the 18 and 21°C treatments ceased after approximately 2 weeks because mortality rates exceeded 50%. Where possible, several individuals from each family (*n* = 3–5) were euthanized prior to *CT*_max_ trials, weighed (g) and measured for total length (mm).

Fish were randomly selected from each family, transferred from holding tanks to *CT*_max_ tanks and covered approximately 24 h before a trial to allow for acclimation to the new tanks. For each treatment, a minimum of 10 individuals per family was used, except for the 15°C treatment (*n* = 4–7), due to high mortality during temperature acclimation. A total of 40–60 fish were used per treatment, and they were placed in small, round open‐topped screened containers taller than water level (258.3 cm^3^) to differentiate individuals. The 4°C treatment was added opportunistically to increase the number of treatments after high mortalities in the 18 and 21°C groups precluded their use in trials, and because insufficient alevin remained from the other five families, only three families were represented in the 4°C group, for which mortality data were not collected. Trials were recorded using ShuttleSoft 3 software (Loligo® Systems, Tjele, Denmark) to log video and temperature data, identify missed observations, and validate the initial observed *CT*_max_. Each container held one to two fish from the same family and was labelled externally by number for identification. The water in the tank was heated at a consistent rate of 0.3°C per minute using six 300‐watt titanium heaters (Finnex, Chicago, Illinois) and two water circulation pumps (Eheim, model 1046 – 5 L/min, Deizisau, Germany) to ensure even heating. Warming rates did not deviate beyond 0.3°C per minute for any of the trials. Dissolved oxygen levels were maintained above 90% throughout the trial. *CT*_max_ was recorded when a fish lost equilibrium or ceased movement with no response to physical stimulation (forceps probing). Once *CT*_max_ was reached, the fish was transferred to a recovery tank at approximately 10°C for 30 min, then euthanized with 400 mg/L tricaine methanesulphonate (TMS) buffered with 800 mg/L sodium bicarbonate.

ShuttleSoft 3 recordings were reviewed to confirm or identify *CT*_max_ values missed during initial observations. For video review, *CT*_max_ was defined as the point when movement ceased, and no response occurred to physical stimulation. Loss of equilibrium was excluded as a criterion because alevin were largely sedentary, and equilibrium loss was inconsistent. Recordings were viewed in reverse to determine the final voluntary movement, and the corresponding temperature was recorded as *CT*_max_. Two independent observers reviewed all videos to minimize bias.

Data analyses were conducted in R (v4.5.1; RStudio Team, 2024). Generalized linear mixed models (GLMMs) were used to test whether acclimation temperature influenced *CT*_max_ and body condition, with family included as a random effect. Model performance was compared using Akaike's Information Criterion (AIC) across Gaussian and Gamma error distributions, with and without outliers (see Tables S1–S3). Fulton's condition factor (FCF) was used to assess variation in alevin body condition across acclimation temperatures. Individual weights were unavailable for all fish because only a random subsample of alevin was weighed to minimize mortality. To examine whether mass affected the relationship between acclimation temperature and *CT*_max_, parametric bootstrapping was used. For each acclimation group, masses were simulated from a normal distribution based on observed means and standard deviations, with sample sizes matching *CT*_max_ trials. Simulated masses were then incorporated into GLMMs (including family as a random effect) across 500 iterations to evaluate robustness.

Bull trout alevin exhibited limited thermal acclimation capacity, with *CT*_max_ plateauing at relatively low acclimation temperatures. *CT*_max_ values increased significantly by 1.5°C between 4°C (24.3°C ± 0.14) and 6°C (25.8°C ± 0.07), increased significantly by 0.9°C between 6 and 9°C (26.7°C ± 0.11), and there was no significant difference between 9 and 12°C (26.9°C ± 0.10) (*F*_4,210_ = 57.93; *p* < 0.001) (Figure 1a). However, *CT*_max_ decreased significantly by 1.0°C between 12 and 15°C (25.9°C ± 0.29). Our results are consistent with previous findings that char *CT*_max_ increases with acclimation temperature and then reaches a plateau; however, the acclimation temperatures where bull trout alevin demonstrate reduced organismal function were much lower than what others report for juvenile brook trout (20–25°C, Morrison et al., 2020) and YOY bull trout (18°C, Best et al., 2025). Moreover, this is the first laboratory study to identify a sublethal thermal acclimation threshold for bull trout that aligns with the 11°C thermal threshold identified for populations in the wild (Best et al., 2025; Isaak et al., 2015; Isaak et al., 2017; Mochnacz et al., 2023). Together, these thermal tolerance thresholds represent critical thermal requirements for this population and highlight the importance of protecting cold‐water habitat.

**FIGURE 1:**
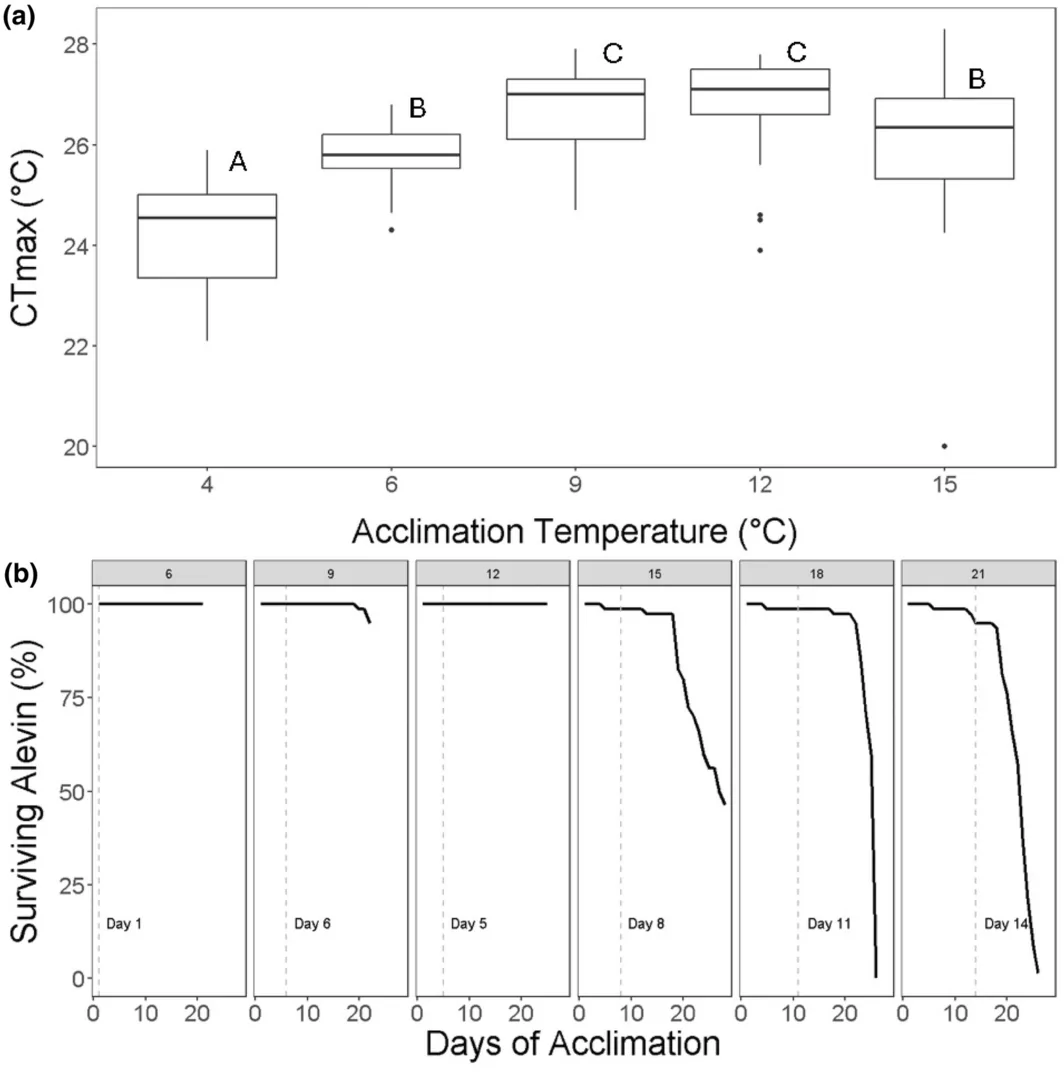
(a) *CT*_*max*_ of bull trout (*Salvelinus confluentus*) alevin across acclimation temperatures ranging from 4 to 15°C (*n* = 40–60 per acclimation group). Horizontal lines denote median values, boxes are 25th to 75th percentile, vertical lines represent adjacent values and dots signify outliers. Letters represent statistical differences (*p* < 0.05). (b) Survival of bull trout alevin through an approximately 30‐day acclimation period at temperatures ranging from 6 to 21°C. Vertical lines denote when the respective acclimation temperature was reached. *N* = 79–80 per acclimation group.

Results demonstrate that *CT*_max_ significantly declines from 12 to 15°C acclimation (Figure 1a) in this population, which implies that at this temperature organismal function declines and individuals cannot tolerate chronic exposure without experiencing adverse effects. Previous research has shown that juvenile brook trout and YOY bull trout *CT*_max_ declines at temperatures higher than 15°C (Best et al., 2025; Durhack et al., 2025; Morrison et al., 2020), providing further evidence that char exhibit intraspecific variation in *CT*_max_ and acclimation capacity across life stages (Dahlke et al., 2020). Interestingly, we also observed high mortality in the warmer acclimation groups starting at 15°C (53.7%) and low mortality (<6%) at 12, 9, and 6°C (Figure 1b). Higher mortality at 15°C demonstrates that this may be a chronic lethal threshold for this life stage, as mortalities began approximately 3 weeks into acclimation, and supports the notion that rearing habitats above 12°C may be unsuitable for bull trout. This upper lethal thermal tolerance of the Smith‐Dorrien Creek bull trout alevin is lower than the 20.9°C upper limit reported for YOY and juveniles elsewhere (Selong et al., 2001). Our results align with the growing consensus that char possess the capacity to thermally adjust to increasing temperatures (Dahlke et al., 2020; Selong et al., 2001). However, Smith‐Dorrien Creek bull trout alevin exhibit the lowest thermal tolerance relative to other YOY and juvenile cold‐water char species in North America (Best et al., 2025; Durhack et al., 2025; Morrison et al., 2020).

In addition to effects on thermal tolerance, acclimation temperature also influenced body condition. FCF was lowest in 12°C (0.42 ± 0.01) and 15°C (0.37 ± 0.01) acclimation groups and significantly higher in the 4°C (0.50 ± 0.01), 6°C (0.56 ± 0.01), and 9°C (0.49 ± 0.01) groups (*F*_5,126_ = 40.90; *p* < 0.05; Table 1). On average, a 1 g increase in mass corresponded to an approximate 5°C rise in *CT*_max_, although the wide 95% confidence interval (−8.73 to 21.3) indicates high uncertainty. Mass was a significant predictor in only 14.6% of simulations, suggesting an inconsistent effect. Reduced FCF at 12°C suggests elevated energetic demands and reduced energy reserves (Benjamin et al., 2020), likely reflecting a mismatch between intake and metabolic demand. This finding is consistent with previous research indicating a reduction in the growth of char species (including bull trout) when subjected to water temperatures beyond optimal ranges (Best et al., 2025; Chadwick & McCormick, 2017; Lonthair et al., 2024; McDermid et al., 2013; Robinson et al., 2010), and may indicate that energy is being reallocated from growth to cope with thermal stress. It is noteworthy that juveniles from this population exhibited thermal stress at the cellular level at temperatures ≥18°C (Best et al., 2025).

**TABLE 1:** Mean total length and mass of bull trout (*Salvelinus confluentus*) alevin across acclimation temperatures from 4 to 15°C. Sample sizes reflect the number of individuals measured per acclimation group.

Acclimation temperature (°C)	Mean total length (mm)	Mean mass (g)	FCF	Sample size
4°C	25.07 ± 0.25	0.079 g ± 0.002	0.50 ± 0.001	15
6°C	24.98 ± 0.11	0.087 g ± 0.002	0.56 ± 0.001	43
9°C	25.20 ± 0.18	0.078 g ± 0.002	0.49 ± 0.001	20
12°C	24.50 ± 0.18	0.061 g ± 0.002	0.42 ± 0.001	20
15°C	24.44 ± 0.20	0.055 g ± 0.002	0.37 ± 0.001	18

*Note*: Values are reported as mean ± standard error.

A limitation of this study is that *CT*_max_ cannot be used an absolute measure of thermal tolerance for wild populations for two reasons. First, the rate of temperature increase to obtain *CT*_max_ in the lab is often greater than typical rates of increase in the wild (Becker & Genoway, 1979). Second, *CT*_max_ values are influenced by the temperatures fish are acclimated to prior to experimentation (Terblanche et al., 2007) and values are not indicative of organ function loss that can occur at temperatures prior to *CT*_max_ during heating (Muñoz et al., 2014). Consequently, when inferring ecological relevance, it is important to compare *CT*_max_ values to thermal tolerance metrics within species or across ecologically similar species (Mayer et al., 2024). Additionally, it is important to consider that the relationship between *CT*_max_ and acclimation temperatures represents the ability of an organism to acclimate (i.e., adjust) across thermal gradients, and when paired with mortality data, can be used to infer sublethal and lethal thermal tolerance thresholds. Lastly, because *CT*_max_ is strongly correlated with other thermal metrics, it can be used to predict lower, more ecologically relevant thermal metrics, based on a population's response to acclimation temperatures (Bartlett et al., 2022; Hasnain et al., 2013; Morrison et al., 2020).

The sublethal and lethal thermal thresholds identified for bull trout alevin from Smith‐Dorrien Creek are critical for conserving this cold‐water species in a warming climate. Sublethal temperatures (12°C) during early development in the wild may limit recruitment by delaying growth and causing a phenological mismatch with prey, resulting in low food supply and mortality. Whereas lethal temperatures (≥15°C) could directly augment population declines. Similar patterns in acclimation capacity across life stages are observed in other salmonids, emphasizing the need for early‐life stage studies to predict climate‐driven survival outcomes (Mayer et al., 2024; Morrison et al., 2020). Our results also support evidence that early stages exhibit lower thermal tolerance and acclimation potential than adults (Dahlke et al., 2020). It is important to recognize results of this study represent the response of one population and therefore should be applied cautiously across the species' range. We encourage others to extend this work to other populations and integrate thermal tolerance results into conservation strategies to mitigate the effects of warming on this societally and ecologically important cold‐water fish.

## AUTHOR CONTRIBUTIONS

Conceptualization: Neil J. Mochnacz, Timothy Bartley and Andrew Chapelsky. Methodology: Brendan Maldonado, Travis Durhack, Andrew Chapelsky and Neil J. Mochnacz. Validation: Brendan Maldonado, Andrew Chapelsky and Travis Durhack. Formal analysis: Brendan Maldonado. Investigation: Brendan Maldonado, Andrew Chapelsky, Raven M. Bennett and Neil J. Mochnacz. Resources: Timothy Bartley and Neil J. Mochnacz. Data curation: Brendan Maldonado, Raven M. Bennett, Sarah E. Glowa and Neil J. Mochnacz. Writing—original draft: Brendan Maldonado and Neil J. Mochnacz. Writing—review and editing: Brendan Maldonado, Travis Durhack, Andrew Chapelsky, Sarah E. Glowa, Analisa Lazaro‐Côté, Raven M. Bennett, Timothy Bartley and Neil J. Mochnacz. Visualization: Brendan Maldonado. Supervision: Timothy Bartley and Neil J. Mochnacz. Project administration: Timothy Bartley and Neil J. Mochnacz. Funding acquisition: Neil J. Mochnacz.

## Supporting information


**Table S1.** Generalized linear mixed model Akaike's Information Criterion (AIC) based on the model distribution and presence or absence of the outlier.
**Table S2.** Estimated marginal means of critical thermal maximum (*CT*_max_) for bull trout (*Salvelinus confluentus*) alevin across acclimation temperatures ranging from 4 to 15°C (*n* = 40–60). Pairwise comparisons indicate significant differences in *CT*_max_ between acclimation groups, with a plateau observed between 9–12°C and a decline at 15°C. Degrees of freedom (df), *t*‐values, and significance in *p*‐value (*p*) are reported for each comparison.
**Table S3.** Pairwise comparisons of bull trout (*Salvelinus confluentus*) alevin body condition (Fulton's condition factor) across acclimation temperatures, with family included as a random effect. Models were repeated 500 times using parametric bootstrapping to ensure robustness. Lower and upper confidence intervals (CI), standard error, and *p*‐values (*p*) are reported for each comparison.

## Data Availability

The data that support the findings of this study are available from the corresponding author upon reasonable request.
