# Supplementary material for: Thermal tolerance and acclimation limits in bull trout (Salvelinus confluentus) alevin
Source: J Fish Biol. 2026 Jul 15;109(4):2017–22. doi: 10.1111/jfb.70580 (PMC13675490; doi:10.1111/jfb.70580)
Supplement: Supplementary file 1 [file JFB-109-2017-s001.docx]

Supporting Information

Our results also show that *CT_max_* declines from the 9-12 °C plateau at 15 °C acclimation. However, there was an outlier of 20 °C in this group that was substantially lower than the group average. Akaike’s Information Criterion (AIC) was compared across several generalized linear mixed models (GLMM), with gaussian and gamma families, with and without the 20 °C outlier to observe the effect of the outlier on the relationship between acclimation temperature and *CT_max_* (Table S1). Removing the outliers lead to a significant reduction in AIC (48), suggesting that the outlier reduced the fit of the model. However, this reduction in AIC is not large enough to justify removing the outliers without careful consideration. We postulate that this outlier likely represents an extreme real case that should be included in the model. There is precedent for an extreme case such as this, as ectotherms have been observed to exhibit intraspecific variation in *CT_max_*, suggesting thermal plasticity can differ between individuals (Chasse *et al*., 2025). Additionally, Morgan et al. (2018) found that individual zebrafish with high innate thermal tolerance had lower acclimation potential than individuals with innately low thermal tolerance. This may explain why an individual bull trout in our experiment expressed substantially lower *CT_max_* compared to other fish in the same acclimation group.

Table S1. Generalized linear mixed model Akaike’s Information Criterion (AIC) based on the model distribution and presence or absence of the outlier.

| **Distribution** | **Outlier Presence** | **AIC** |
| --- | --- | --- |
| Gaussian | Present | 608.95 |
| Gaussian | Absent | 560.80 |
| Gamma | Present | 612.46 |
| Gamma | Absent | 557.52 |

Table S2. Estimated marginal means of critical thermal maximum (*CT_max_*) for bull trout (*Salvelinus confluentus*) alevin across acclimation temperatures ranging from 4 – 15 °C (n = 40-60). Pairwise comparisons indicate significant differences in *CT_max_* between acclimation groups, with a plateau observed between 9–12 °C and a decline at 15 °C. Degrees of freedom (d.f.), t-values, and significance in p-value (p) are reported for each comparison.

| **Pairwise Comparison** | **d.f.** | ***t*** | **p** |  |
| --- | --- | --- | --- | --- |
|  |  |  |  |  |
| T4 °C - T6 °C | 215 | -8.137 | < 0.001 |  |
| T4 °C - T9 °C | 213 | -12.275 | < 0.001 |  |
| T4 °C - T12 °C | 212 | -13.789 | < 0.001 |  |
| T4 °C - T15 °C | 202 | -5.618 | < 0.001 |  |
| T6 °C - T9 °C | 213 | -4.766 | < 0.001 |  |
| T6 °C - T12 °C | 213 | -6.151 | < 0.001 |  |
| T6 °C - T15 °C | 214 | -0.249 | 0.999 |  |
| T9 °C – 12 °C | 212 | -1.164 | 0.772 |  |
| T9 °C - T15 °C | 215 | 2.889 | 0.034 |  |
| T12 °C - T15 °C | 215 | 3.713 | 0.002 |  |

Table S3. Pairwise comparisons of bull trout (*Salvelinus confluentus*) alevin body condition (Fulton’s condition factor) across acclimation temperatures, with family included as a random effect. Models were repeated 500 times using parametric bootstrapping to ensure robustness. Lower and upper confidence intervals (CI), standard error, and P-values (P) are reported for each comparison.

| **Pairwise Comparison** | **Lower CI** | **Upper CI** | **Standard Error** | **P** |
| --- | --- | --- | --- | --- |
| T4 °C - T6 °C | 0.008 | 0.109 | 0.012 | 0.014 |
| T4 °C - T9 °C | -0.072 | 0.043 | 0.014 | 0.979 |
| T4 °C - T12 °C | -0.143 | -0.028 | 0.014 | 0.001 |
| T4 °C - T15 °C | -0.19 | -0.071 | 0.014 | > 0.001 |
| T4 °C - T18 °C | -0.17 | -0.049 | 0.015 | > 0.001 |
| T6 °C - T9 °C | -0.119 | -0.027 | 0.011 | > 0.001 |
| T6 °C - T12 °C | -0.19 | -0.098 | 0.011 | > 0.001 |
| T6 °C - T15 °C | -0.237 | -0.142 | 0.012 | > 0.001 |
| T6 °C - T18 °C | -0.218 | -0.118 | 0.012 | > 0.001 |
| T9 °C – 12 °C | -0.125 | -0.018 | 0.013 | 0.003 |
| T9 °C - T15 °C | -0.171 | -0.061 | 0.013 | > 0.001 |
| T9 °C - T18 °C | -0.152 | -0.038 | 0.014 | > 0.001 |
| T12 °C - T15 °C | -0.1 | 0.01 | 0.013 | 0.172 |
| T12 °C - T18 °C | -0.081 | 0.033 | 0.014 | 0.824 |
| T15 °C - T18 °C | -0.037 | 0.079 | 0.014 | 0.899 |

**References**

Chasse, S. D., Stewart, E. M. C., & Raby, G. D. (2025). Repeatability of critical thermal maximum (*CT_max_*) in two freshwater ectotherms across contexts. *Journal of Thermal Biology,* **127**, 104049. https://doi.org/10.1016/j.jtherbio.2025.104049

Morgan, R., Finnøen, M. H., & Jutfelt, F. (2018). *CT_max_* is repeatable and doesn’t reduce growth in zebrafish. *Scientific Reports,* **8**, Article 7099. https://doi.org/10.1038/s41598-018-25593-4
